# Visual completion from 2D cross-sections: Implications for visual theory and STEM education and practice

**DOI:** 10.1186/s41235-016-0010-y

**Published:** 2016-09-22

**Authors:** Kristin Michod Gagnier, Thomas F. Shipley

**Affiliations:** 1 Science of Learning Institute, Johns Hopkins University, 3400 N. Charles Street, Baltimore, MD 21218-2685 USA; 2 Spatial Intelligence and Learning Center, Department of Psychology, Temple University, Philadelphia, PA 19122 USA

**Keywords:** Perception of 3D volumes, Amodal completion, Spatial reasoning, STEM education

## Abstract

Accurately inferring three-dimensional (3D) structure from only a cross-section through that structure is not possible. However, many observers seem to be unaware of this fact. We present evidence for a 3D amodal completion process that may explain this phenomenon and provide new insights into how the perceptual system processes 3D structures. Across four experiments, observers viewed cross-sections of common objects and reported whether regions visible on the surface extended into the object. If they reported that the region extended, they were asked to indicate the orientation of extension or that the 3D shape was unknowable from the cross-section. Across Experiments 1, 2, and 3, participants frequently inferred 3D forms from surface views, showing a specific *prior* to report that regions in the cross-section extend straight back into the object, with little variance in orientation. In Experiment 3, we examined whether 3D visual inferences made from cross-sections are similar to other cases of amodal completion by examining how the inferences were influenced by observers’ knowledge of the objects. Finally, in Experiment 4, we demonstrate that these systematic visual inferences are unlikely to result from demand characteristics or response biases. We argue that these 3D visual inferences have been largely unrecognized by the perception community, and have implications for models of 3D visual completion and science education.

## Significance

Practitioners of science, technology, engineering, and mathematics (STEM) disciplines such as radiologists, geologists, and surgeons often have to make inferences about the three-dimensional (3D) structure of objects (organs, rocks, tumors) from two-dimensional (2D) surface views such as magnetic resonance imaging (MRI) slices or outcrops of rock. These inferences represent a challenging visual problem that has not been explored in the literature on amodal visual completion. Additionally, these inferences can be challenging for students, and understanding why they are difficult has the potential to inform education. In this paper, we present data that suggest specific priors in how observers infer 3D structures from 2D cross-sections; these priors influence both the accuracy of observers’ inferences and recognition of when accurate inferences are not possible. These priors have implications for theories of visual processing and also in science education for how to develop effective pedagogical approaches to teaching students to make inferences about the 3D structures from 2D cross-sections.

## Visual Completion from 2D Cross-Sections: Implications for Visual Theory and STEM Education and Practice

**Fig. 1:**
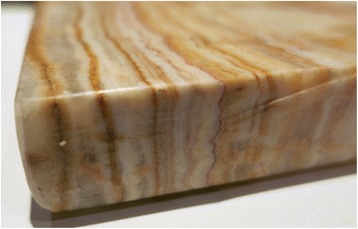
Two orthogonal faces of planar layers of marble. By inspecting both the top and side, it is possible to infer that the rust-colored forms that appear as lines on each face are the visible expression of 3D planar layers that extend through the object

If you inspect the rust-colored lines on each side of the rock in Fig. 1, no doubt you will have the impression of planar layers of mineral in the marble. Observers seem to have no difficulty connecting the similar-colored lines on each side, and making an inference about how they extend into the rock. Notice that we use the term *inference* here because one cannot actually see the planar form inside the rock. This may seem like a trivial achievement. In this case, accurately inferring the 3D shape of the layers from the surface features is possible because two sides of the rock are visible, so the internal structure may be inferred by filling in the plane between the lines. However, consider the problem if only a single side were visible, as is the case in a single cross-sectional view of any object. In this case, the single planar layer of marble would appear as a line on the surface; however, the line would provide no information about the orientation of the internal layer.

**Fig. 2:**
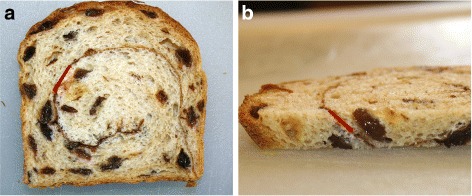
Image of a slice of raisin bread. **a** Participants indicated if the region highlighted by the *red line* was present on the surface or extended into the object. **b** The inside of the bread showing the angle at which the highlighted region extends into the object (indicated with a *red line*). Participants never saw this view

To illustrate, we provide an example outside of geology and invite the reader to consider the slice of bread shown in Fig. 2a. Most people report a clear impression that the cinnamon layer extends straight back into the slice, often arguing, “How could it be otherwise; surely the bread was baked with a layer that extended from one end to the other.” This logic is faulty, as it assumes (a) completely regular dough thickness during the rolling of dough and cinnamon, (b) perfect alignment of the shaped dough with the pan, and (c) completely isotropic rising. More importantly, in this case, it is incorrect. The dip^1^ of the cinnamon is shown in Fig. 2b; the cinnamon extends into this slice at a dip angle of 60°, not 90°.

Here we present work on a class of 3D visual inferences that has not received much attention in the visual perception community – *inferring the 3D interior structure of an object from surface features and possible constraints on these inferences*. This visual task is important for many science, technology, engineering, and mathematics (STEM) disciplines in which 3D structures are measured or displayed using a series of cross-sections. (For example, a radiologist infers the 3D structure of a tumor from MRI scan slices, and a geologist infers 3D structures in the Earth from outcrops visible on the surface.) This task is challenging for students (Kali & Orion, 1996) and experts (Bond, Lunn, Shipton, & Lunn, 2012) alike. Understanding visual inferences about 3D structures can offer insights into principles of visual processing and has implications for STEM education and practice. Our aims are to begin to characterize visual inferences about 3D structure from surface features and to highlight the similarity between this inference and amodal completion (Kanizsa, 1979; Michotte, Thinès, & Crabbé, 1964).

## Background

A central problem in midlevel vision is to understand how the visual system uses light projected onto a 2-dimensional (2D) surface to represent 3-dimensional (3D) properties of the world. Our world is 3D, and thus we have to mentally represent and process the 3D structure of objects. Yet, the retinal image is flat, so the 3D structure of an object has to be inferred by the visual system. Much of the work on this problem has been focused broadly on the question of how spatial relations are recovered from the 2D retinal projection. Seminal work on this problem has identified how 3D shape is inferred from stereoscopic disparity (Marr & Poggio, 1976), edges (Marr & Hildreth, 1980), contour shape (Marr & Nishihara, 1978; Ullman, 1989), shading (Buelthoff & Yuille, 1991; Hayward, 1998), silhouette (Koenderink & Van Doorn, 1991), motion (i.e., the kinetic depth effect; Wallach & O’Connell, 1953), and textural gradients (Gibson, 1950), collectively referred to as *shape-from-X* (Buelthoff & Yuille, 1991).

The problem of inferring the 3D shape from a cross-section is similar to other 3D shape-from-X problems where observers have to make inferences about the 3D shape from limited perceptual information. Despite the inherent ambiguity of structures visible on a cross-sectional surface, observers may nevertheless have clear and systematic impressions of the shape and orientation these structures take as they extend into the object. The possibility that observers have clear and systematic impressions of how regions extend into cross-sections, or *priors*, first came to our attention in discussions with geologists, who noted that their students often reported that geological structures seen in an outcrop (a cross-section of rock) extended *straight back* into the rock (Kali & Orion, 1996). The purpose of the work presented in this paper is to examine the generality and implications of this observation.

We borrow the term *prior* from the Bayesian framework, which models decision-making under uncertainty. This framework has been applied successfully to understanding the role of prior knowledge in visual perception, as well as perceptual illusions and constancies (Kali & Orion, 1996). This work proposes that experience on a personal or evolutionary time scale supports unconscious statistical inferences (Helmholtz, 1867) about the probability of the environment’s being a particular way, given specific sensory input. For example, in apparent motion, there is a specific prior to infer a straight path between objects that successively appear in different locations, unless the objects are biological, where curved paths may be more likely (Heptulla-Chatterjee, Freyd, & Shiffrar, 1996). In such a case, priors may reveal something about the statistical likelihood of objects or events in the environment.

Across four experiments, we examined visual inferences from structures visible only in cross-sectional views (e.g., the rust-colored lines in Fig. 1 and the cinnamon swirl in Fig. 2). We showed undergraduate psychology students photographs (Experiments 1 and 2) or 3D objects (Experiment 3) where a single surface was visible and asked for their impression of how a specific region extended into the object. We examined whether (a) observers tended to infer 3D forms from single surface views or whether this phenomenon was restricted to a few rocks or geology students, (b) there is a prior to infer regions visible in a cross-section as extending back into the object at 90°, (c) observers can correctly infer the 3D structure if given sufficient information, and (d) the visual inferences made from cross-sections occur in spite of world knowledge and thus may be similar to the visual inferences seen in amodal completion (Kanizsa & Gerbino, 1982; Michotte et al., 1964), where structures that are not visible are inferred from visible structures.

To preview our results, in Experiments 1, 2, and 3, observers did indeed report regions in cross-sections as extending straight back into the object at 90°. However, in Experiment 2, when observers were given sufficient information, they were generally correct in their inferences and thus did not show evidence of this prior. Finally, in Experiments 3 and 4, we show that this prior is not adjusted by memory or beliefs, suggesting similarities with amodal completion. We argue that this prior to report regions as extending straight back is revealing about how the perceptual system processes information about the interior of objects from information on the object’s surface and is relevant to STEM education where students learn about interior structures from cross-sections.

## Experiment 1

The purpose of Experiment 1 was to describe naïve observers’ reports of the internal structure of objects inferred from surface patterns. Do observers (a) report 3D forms, and, if so, (b) how do they report those regions as continuing into the object? Observers viewed photographs of cross-sections of everyday objects (rocks, wood, food) and indicated whether a region highlighted with a red line (shown in Fig. 2a) was present only on the surface or whether it extended into the object. If they thought it extended into the object, they used a bar attached to an inclinometer to show the angle of extension. If participants tended to infer regions in a cross-section as extending straight back in three dimensions, then we would expect dip estimates to cluster near 90°. If inferences of cross-sections are not systematic, then we would expect to see a wide range of estimates of extension (e.g., some estimates at 45°, some at 170°).

Anticipating there might be some individual differences, we sought to determine whether there was a relationship between the estimated dip angle and performance on measures of spatial reasoning about perspective and orientation of 3D forms. We hypothesized that observers who performed better at measures of spatial reasoning might be more likely to recognize that one cannot know the true 3D shape from a single cross-sectional view.

### Methods

#### Participants

Participants were 30 Temple University undergraduates (19 females) fulfilling a requirement for an introductory psychology course.

#### Stimuli

The stimuli consisted of 17 color photographs cross-sections of common objects such as food, wood, and rocks, as shown in Fig. 2a. One practice image (a Swiss roll) and 16 experimental images were used. Stimuli fell into three categories: (a) *biological* [fruits (n = 3); wood (n = 2); vegetable, fish, and meat (n = 2)], (b) *geological* [rocks (n = 4)], and (c) *analogues to igneous rocks*—these are food products that were originally liquid and are now solid (blue cheese, chocolate with almonds, and cinnamon bread). The images were approximately 25 × 18 cm when presented on the screen.

These categories were selected because the internal structure of the objects ranged from highly structured and constrained by the environment (e.g., wood grain) to relatively unconstrained (e.g., minerals in rock), and thus the angle of extension into the object is either predictable within a certain range or completely unpredictable. For example, the internal structure of wood is constrained by the environment. As tree structures are generally concentric cylinders, the extension into a block of wood is a function of the angle of the cut relative to the cylinders. The internal composition of rocks can be structured, but the orientation of a mineral’s surface relative to the cutting plane is essentially arbitrary, and thus the 3D structure is unpredictable from a single cross-section. This is also true for geologically analogous stimuli such as nuts in chocolate—the orientation of the nut relative to the orientation of the cut is arbitrary.

These objects were chosen with two additional constraints: (a) that we were physically able to slice each object and measure the true angle at which each highlighted region extended into the object, and (b) that we included a variety of objects that might be familiar to participants.

#### Apparatus

**Fig. 3:**
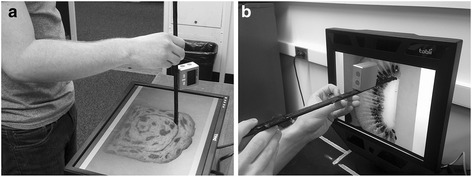
**a**: The display used in Experiment 1. Participants used the black bar to indicate the orientation at which the highlighted region extended into the object. **b**: The display used in Experiment 2

Stimuli were presented on a 20-inch Dell monitor. As shown in Fig. 3a, the monitor was positioned parallel to the ground.

#### Procedure

Participants were tested individually in a well-illuminated room. They viewed each picture while standing with both eyes open and positioned over the center of the monitor (as shown in Fig. 3a). Participants were told that we were interested in their opinions of how regions continue in three dimensions. They were told that sometimes they would see pictures where they might have a strong sense that a region continued and sometimes they might have a sense that something was present only on the surface. To illustrate these, students were shown a cross-section of the front of a Swiss roll (a filled pastry with alternating layers of chocolate cake and cream) and crayon marks on paper. All students reported the layers of the Swiss roll as extending into the object while the crayon marks were present on the surface.

Observers were then shown 16 pictures. For each, they indicated whether the region indicated with a red line was present only on the surface (like the crayon marks on the paper) or extended into the object (like the Swiss Roll). If they thought the region extended into the object, they indicated the orientation using a stainless steel bar with an attached inclinometer (to measure angle). Participants placed the end of the bar on the red line and then moved the bar to indicate the angle. The 0° was defined relative to the ground plane (i.e., if the bar was angled straight down, as shown in Fig. 3a, the angle measurement was 90°). After this, they reported their confidence in their response on a 5-point scale (5 indicates “extremely confident”). Prior to viewing the 16 pictures, participants practiced orienting the bar on the image of the Swiss roll.

To be sure that there were no differences in the estimates based on the orientation of the picture, participants viewed all 16 pictures in their original orientation and rotated 180°. This allowed us to calculate any bias due to body position relative to the image. Finally, participants were shown the pictures a third time and asked to identify each picture. For any response given a confidence rating of 0 or 1, we further probed the participants’ uncertainty by asking them which of the following reasons best described their confidence rating: (a) they have no idea what the orientation could be, (b) the orientation is not knowable from the picture, or (c) there could be a range of possible orientations at which the region extended into the object. After this, participants completed three measures of spatial reasoning.

#### Measures of Spatial Reasoning

**Fig. 4:**
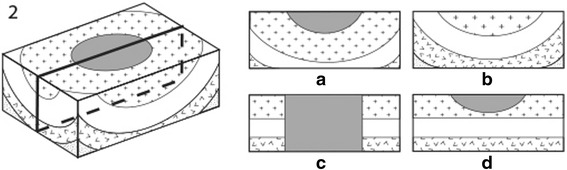
An example of a problem from the Geologic Block Cross-Sectioning Test and corresponding answer choices (**a**-**d**). The correct answer is A

The Geologic Block Cross-Sectioning Test (GBCT; Ormand et al., 2014) is a measure of visualizing volumetric forms from cross-sections. Participants were given 8 minutes to complete 14 problems in which they had to select the cross-section that resulted from a pictured cut into a geologic block diagram (as shown in Fig. 4).

The Object Perspective Taking Test (Kozhevnikov & Hegarty, 2001) is a test in visualizing the locations of objects when seen from a specific perspective. A configuration of seven objects is shown, and participants imagine standing at the position of one object, facing another object, and they indicate the direction to a third object. Participants had 5 minutes to complete 12 questions, and the dependent measure is angular error.

The Peters and colleagues (1995) paper-and-pencil version of the Vandenberg and Kuse (1978) Mental Rotation Test measures skill in visualizing objects after they are rotated. Observers viewed five line drawings of 3D forms similar to those used by Shepard and Metzler (1971). The target form is on the left, and four answer choices are presented on the right. For each problem, participants identify the two choices that are identical but rotated versions of the target form. The test has 2 parts with 12 problems each, and participants were given 3 minutes to complete each half.

#### Unbiased Estimate Measurement

To calculate the participant’s unbiased estimate for each picture, we combined the two estimates by calculating the average of the first estimate and 180° minus the second estimate (when the picture was rotated 180°). Overall, participants exhibited a bias of 4.1° toward their body.

### Results and Discussion

Participants were fairly confident that the 3D form could be determined from a cross-sectional view. In 74 % of the trials, they reported the region as “going into” the object and gave an estimate of dip with a mean confidence of 3.2 (SD 1.1). There was variation across images. Dip estimates were given most often for the salmon cross-section (97 % of the trials) and least often for the papaya cross-section (37 %).

**Fig. 5:**
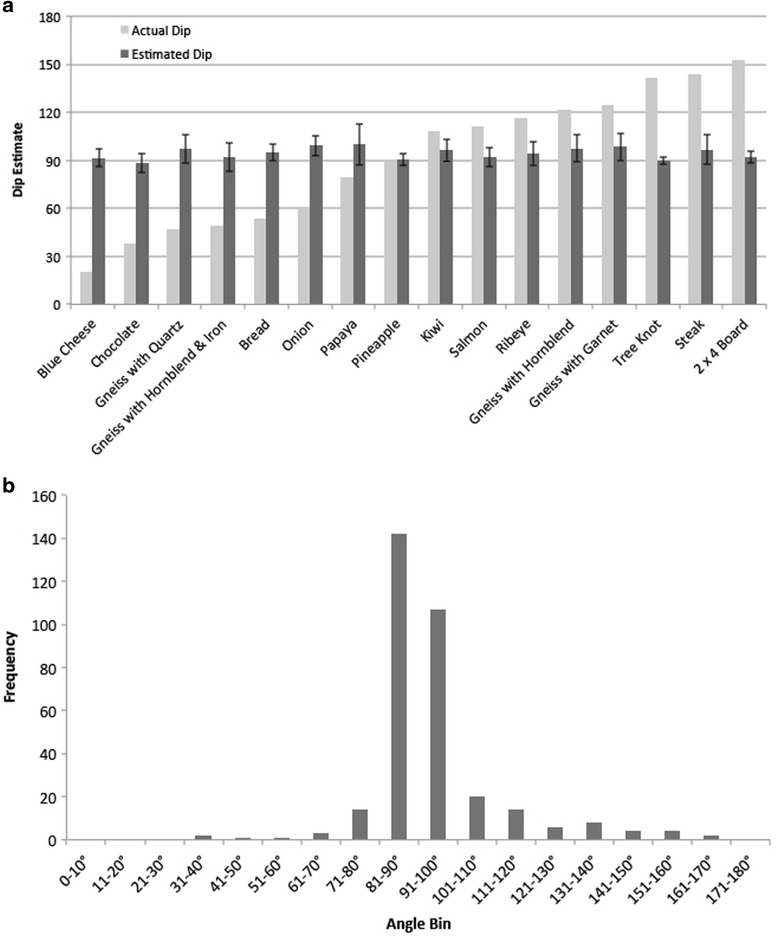
**a** The mean dip estimates (*dark gray*) for each picture along with the correct dip angle for each picture (*light gray*) in Experiment 1. Note that dip angle is defined relative to the plane of the picture. A report of a region as continuing “straight back” would be a dip estimate of 90°. **b** Frequency distribution of responses that fell within 10° angle bins in Experiment 1

Figure 5a shows the true dip angle (how the highlighted region actually extended into the object) and participants’ mean dip estimate for each picture. Although there is a wide range of true dip angles, participants reported regions as extending *straight back* into the object. Fifteen of the 16 pictures have mean dip estimates that are not significantly different from 90°.^2^ Figure 5b shows the frequency distribution of dip estimates across all pictures. The estimates cluster near 90°, with relatively little variability: 76 % of responses were within 10° of 90°. When participants reported the regions as extending into the object, they said the regions extended straight back.

Next, we examined performance on our three measures of spatial reasoning. The mean number correct on the GBCT was 3.9 (SD 2.0) out of 14. The mean angular error for the Object Perspective Taking Test was 52.2 (SD 26.3).^3^ The mean number correct on the Mental Rotations Test was 7.4 (SD 3.3) out of 24. Performance on the spatial measures did not predict the mean dip angle estimates (*R* ^2^ = .005, *F* < 1). This is perhaps not surprising, given the limited range of reported dip angles; most regions were reported as extending straight back into the objects.

In sum, the responses indicated a prior to infer structures visible in a single cross-section extending straight back into the object at 90°. This was not limited to rocks, but occurred for familiar objects.

## Experiment 2

In Experiment 2, we further probed the nature of the prior. We reasoned that perhaps observers knew that the orientation of an extension was unknowable from a single cross-sectional view, but because they were given only two answer choices (surface vs. extends in), they were prevented from expressing this knowledge. To address this, participants in Experiment 2 selected one of the following answer choices for each picture: (a) the region extends into the object, and I can show you the angle of extension; (b) the region is present only on the surface; (c) the region extends in, but from the picture you cannot know how it extends in; and (d) from the picture, you cannot tell if the region extends in or is on the surface (i.e., the answer is unknowable). As in Experiment 2, we examined whether there was a relationship between spatial reasoning performance and how likely a participant was to understand the uncertainty of a cross-sectional view. We also changed the orientation of the image to be sure that the effect observed in Experiment 1 was not dependent on the particulars of the viewing perspective. In Experiment 2, participants viewed the screen oriented perpendicular to the ground and indicated their dip estimates from this position (as shown in Fig. 3b).

**Fig. 6:**
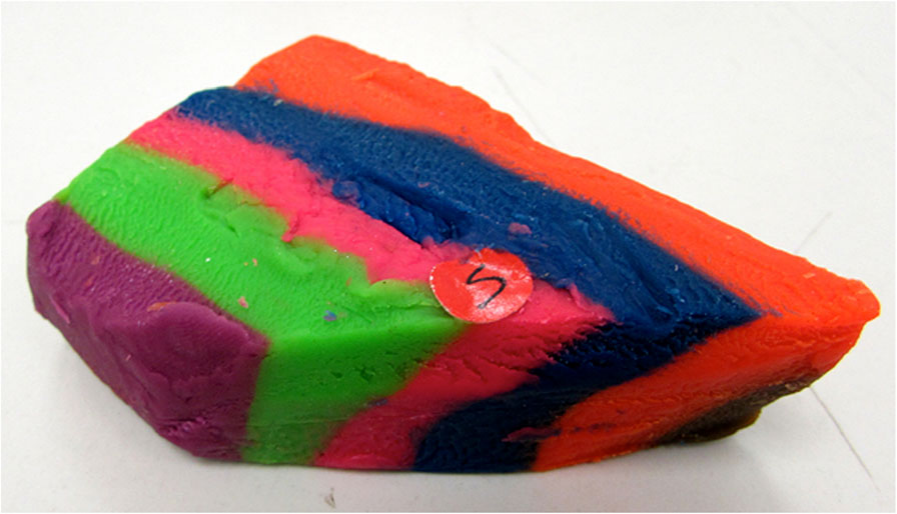
One of the Play-Doh models used in Experiment 2. For each, a layer was indicated with a colored and numbered sticker. Participants oriented a bar attached to an inclinometer and used this to show the angle at which the layer extended into the model

Finally, we included two additional types of stimuli. First, we added five additional pictures in which the region was present only on the surface to serve as a check to ensure participants were using our dependent measure correctly. Second, after giving their responses to the pictures, participants viewed 3D models of layers of Play-Doh (as shown in Fig. 6). As observers could see how a region shown on the top extended in three dimensions by also looking at the cross-section on the side, we could determine if participants could correctly infer the orientation of extension when given sufficient information to solve the problem.

### Methods

#### Participants

Participants were 55 Temple University undergraduates (29 females) fulfilling a requirement for an introductory psychology course.

#### Stimuli

The 16 stimuli from Experiment 1 were used with the addition of 5 new pictures that presented regions present only on the surface (e.g., moss on wood, markers on paper, paint on wood). Participants also viewed eight Play-Doh models (see Fig. 6). These were Play-Doh models consisting of different colored layers that had been transformed in various ways, such as folded or tilted. The Play-Doh models ranged in size from 2 × 6 × 4 cm to 5 × 8 × 6 cm (height × width × depth).

#### Apparatus

Stimuli were presented on a 17-inch Tobii 1750 (Tobii, Stockholm, Sweden) eye tracker screen (eye tracking data were not collected for this experiment). As shown in Fig. 3b, participants made their estimates sitting with their line of sight parallel to the ground and were seated approximately 45 cm away from the screen.

#### Procedure

The procedure was similar to that in Experiment 1, with the following exceptions. Participants viewed 21 pictures in an original and 180° rotation while seated at a desk and the monitor was positioned in front of them. For each picture, they selected one of the following answer choices and then indicated their confidence: (a) the region extended into the object and they could use a bar to show the angle of extension; (b) the region was present only on the surface; (c) the region extended into the object, but from the picture they could not know how; or (d) from the picture, one cannot know whether the region extends into the object or is present only on the surface (i.e., answer is unknowable). Observers then viewed eight Play-Doh models in physical space (not on a computer screen). For each model, a layer was indicated with a small sticker (see Fig. 6), and observers oriented a bar to show how the layer extended into the model.

### Results and Discussion

As in Experiment 1, participants were fairly confident that the 3D orientation of the region could be determined from a single cross-sectional view; mean confidence was 3.7 (SD .97). Even when participants were explicitly asked if the answer could be determined from the information in the picture, they reported the region as “going in” on 56 % of trials (dip estimates were given on 37 % of the trials, and on 19 % of the trials participants reported that the region “extended into the object but from the picture one cannot know how”). Overall, fewer dip estimates were given in Experiment 2 than in Experiment 1. Estimates were most likely for the cinnamon bread (71 % of the trials) and least likely for gneiss with garnet (11 % of the trials). Participants reported that the answer could not be determined in only 7 % of the trials. On average, the images of material on surfaces (e.g., moss on wood) were reported as extending into the object in only 8 % of trials, suggesting that participants understood and used our rating scale appropriately.

**Fig. 7:**
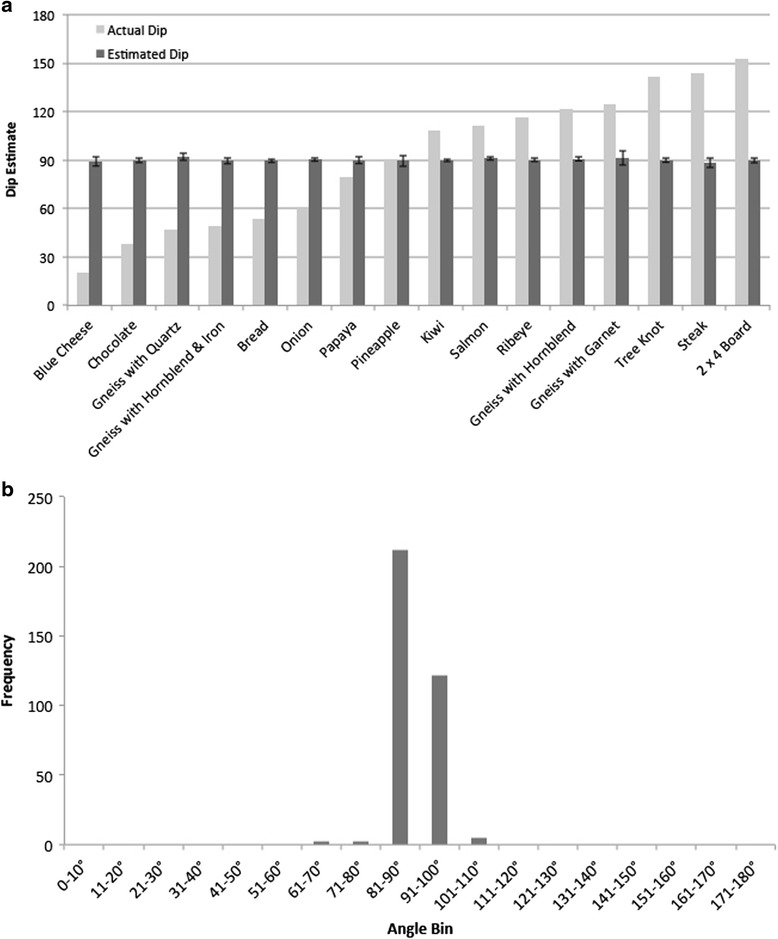
**a** The mean dip estimates (*dark gray*) for each picture along with the correct dip angle for each picture (*light gray*) in Experiment 2. **b** Frequency distribution of responses that fell within angle bins in Experiment 2

We replicated the dip angle findings of Experiment 1; participants consistently reported regions extending straight back into the object. Figure 7a shows the mean dip estimate and the correct dip angle for each picture. As in Experiment 1, there is a wide range of true dip angles; yet, participants reported the regions as extending straight back into the object. As can be seen in the figure, all of the 16 pictures have mean estimates that do not differ significantly from 90°. Figure 7b shows the frequency distribution of dip estimates across all pictures. There was little variability in dip; 97 % of the responses fell between 80° and 100°.

**Fig. 8:**
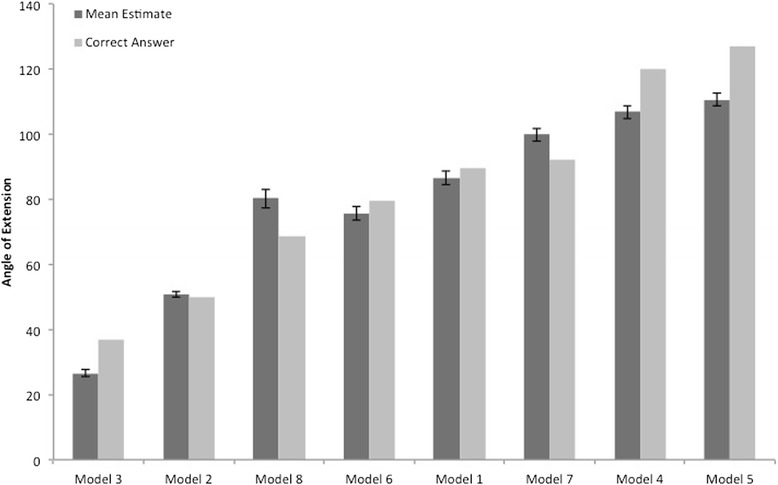
The mean dip estimates (*dark gray*) along with the correct dip angle for each picture (*light gray*) for the Play-Doh models in Experiment 2

To determine if correct visual inferences can be made when given sufficient information, we examined performance on the Play-Doh models. The mean dip estimate and the correct answer are shown in Fig. 8. While there is some variation (i.e., participants are not perfectly correct at inferring the 3D structure when two sides are visible), 51 % of the responses fell with 10° of the correct answer and 75 % of the responses fell within 20° of the correct answer. Thus, when two sides were visible, observers made fairly accurate inferences. Performance on this task was likely not perfect, because interpolating the angle of an edge defined by the intersection of two planes is challenging (Pani, William, & Shippey, 1998).

The mean number correct on the GBCT was 4.9 (SD 2.6) out of 14. The mean number correct on the Mental Rotations Test was 8.0 (SD 5.3) out of 24. The mean angular error for the Object Perspective Taking Test was 49.5 (SD 27.4). We hypothesized that students who had high spatial reasoning performance might be more likely to recognize that the 3D shape is unknowable from a 2D sectional view. Three participants were excluded from these analyses because they did not complete all spatial measures. We conducted a stepwise multiple regression analysis with normalized performance on our three spatial measures. At Step 1, GBCT performance entered into the regression and was significantly related to number of estimates [*F*(1,50) = 8.2, *p* < .01, *R* ^2^ = .14], indicating that 14 % of the variance of the number of estimates was accounted for by GBCT performance. Neither mental rotation (*t* = .77, *p* = n.s.) nor perspective taking (*t* = .63, *p* = n.s.) performance entered into the analysis at Step 2, suggesting that neither test accounted for significant variance in the number of estimates (this was true even when these variables were entered in first). This indicates that observers who are better able to infer 3D forms from diagrams (as assessed by the GBCT) are more likely to know that additional information is needed to infer the orientation of a cross-sectional region. Furthermore, recognizing the ambiguity of a single cross-section is not related to mental rotation or perspective-taking skill. The lack of a relationship here suggests that participants are not simply reasoning about this task, and is consistent with the visual inference from a cross-section being a perceptual phenomenon.

## Experiment 3

Together, the results of Experiments 1 and 2 suggest that observers have a prior to infer regions as extending straight back into the object. Is the prior based on memory for the object, or is it based on the visual information present at the surface? To address this question, we showed observers 3D Play-Doh models where the top surface was visible and asked how a region extended into the model. We then showed them the correct answer (by revealing the side) and then covered the correct answer and asked if their reports of the dip reverted to their original report or conformed to their memory for the correct extension. If the prior observed in Experiments 1 and 2 is not the result of memory, this suggests it may be similar to the visual inferences seen in amodal completion. Although there is debate about whether high-level processes are involved in amodal completion (Lee & Vecera, 2005; Rauschenberger, Peterson, Mosca, & Bruno, 2004), most work has shown that amodal completion processes are perceptual in nature and not influenced by knowledge (Kanizsa, 1979; Pratt and Sekuler, 2001).

**Fig. 9:**
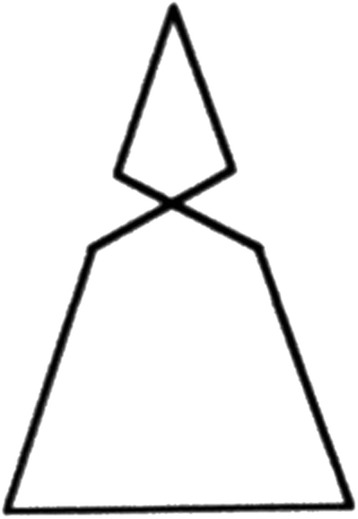
The original form used by Michotte and colleagues (1991) to study amodal continuation. If a finger is placed over the X, participants report a complete isosceles triangle in spite of their prior knowledge about the structure

To illustrate the lack of an effect of prior knowledge on amodal completion, we invite the reader to place a finger over the X portion of Fig. 9. Most observers report *seeing* a complete isosceles triangle, even though the observer *knows* this to be false. Michotte and colleagues (1991) argued that memory does not influence perception, and thus amodal completion is a perceptual phenomenon in which the visual inference is hardwired (e.g., cognitively encapsulated; Fodor, 1983). The purpose of Experiment 3 was to examine if the prior reported here occurs in spite of or as a function of knowledge about the 3D properties of the specific object.

**Fig. 10:**
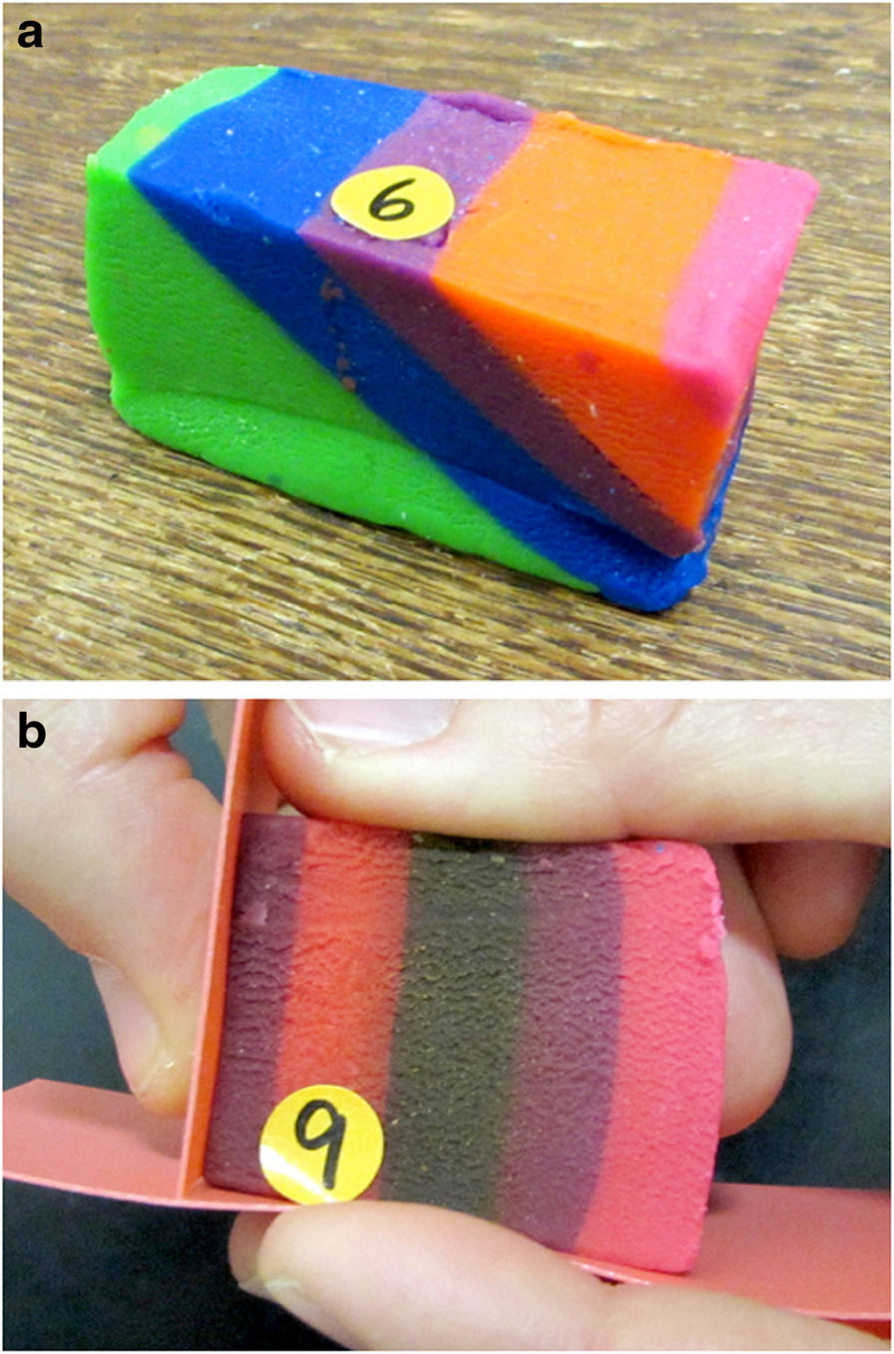
**a** An example of the Play-Doh models used in Experiment 3. **b** Each model was shown originally so only the top was visible to participants. For each, a layer was indicated with a colored and numbered sticker. Participants oriented a bar to show the angle at which the layer extended into the model

Participants viewed 13 Play-Doh models consisting of colored layers of Play-Doh in different 3D orientations in which only the top was visible (as shown in Fig. 10b). We asked observers how a layer of Play-Doh extended into the model. We then showed the correct answer (akin to showing the X region in Fig. 9) and then covered the side again and asked participants whether the layer had the same orientation as it had originally or if the orientation now conformed to their memory of the orientation. Before the experiment, participants were shown the Michotte triangle as a demonstration of seeing something other than what one knows is true.

If the prior observed in Experiments 1 and 2 reflects participants’ knowledge of the object, then we would expect participants to switch their reports to be in line with their knowledge of the correct answer. If, however, this prior occurs in spite of knowledge (and is thus similar to amodal completion), then we would expect participants to report their original experience even though they know the correct answer.

### Methods

#### Participants

Participants were 42 Temple University undergraduates (31 females) fulfilling a requirement for an introductory psychology course. Two participants were excluded from the analysis for not following instructions.

#### Stimuli

The stimuli consisted of 13 Play-Doh models. As shown in Fig. 10a, the models consisted of different colored layers that had been folded or tilted. The models ranged in size from 2 × 3.5 × 2.5 cm to 3 × 6 × 4 cm (height × width × depth).

#### Procedure

Participants viewed 13 Play-Doh models and were asked to distinguish between what they saw when they looked at them and what they knew to be true about them. They were told that what they see and what they know *might* be the same or could be different. We assured participants that there were no right or wrong answers other than not reporting what they experienced.

As an illustration of what was meant by “distinguishing between seeing and knowing,” participants were asked what they saw when shown the Michotte and colleagues (1991) triangle, with a piece of paper taped over the middle that occluded the X region. Every participant reported seeing a triangle. The paper was then removed, and participants were asked whether the twist in the triangle was what they were expecting to see. Then the paper was placed back over the X region, and participants indicated if their perception was the same as it was originally or whether their perception changed to reflect what they *knew* was under the paper. All participants reported seeing the original triangle.

After this demonstration, participants were shown each Play-Doh model where only the top was visible. The sides of the model were covered with small sheets of opaque plastic (as shown in Fig. 10b). For each model, a layer was labeled with a small sticker and participants were asked about that layer. First, they were asked whether they perceived the layer to be present only on the surface or as extending into the model. If observers perceived the layer as extending in, they oriented a bar attached to an inclinometer to show the angle of extension. An experimenter then removed the plastic covering to show the actual angle at which the layer extended into the model. The side was then covered back up, and the observer was asked whether they perceived it as they had originally (like the triangle) or whether now their perception had changed as a result of new information (unlike the triangle). If they had reported the layer as extending in, they oriented the bar a second time to show the angle of extension.

### Results and Discussion

**Fig. 11:**
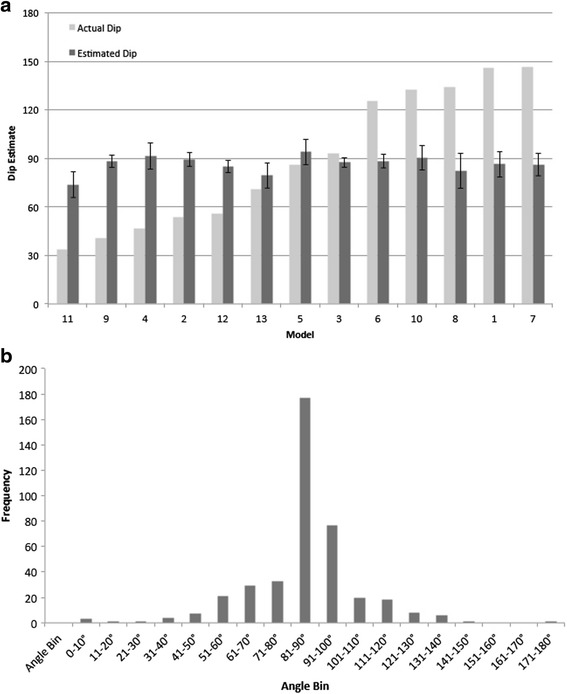
**a** The mean dip estimates (*dark gray*) when viewing the top of the model only and correct dip angle for each model (*light gray*) in Experiment 3. **b** Frequency distribution of responses that fell within angle bins in Experiment 3

Participants reported the layer as extending in in 78 % of the trials. Figure 11a shows the correct dip angle and the mean dip estimate given for each model when only the top was visible. Participants tended to report the layer as extending straight back into the model. Although there is greater variation in the responses to these 3D models, it replicates the prior observed in Experiments 1 and 2. As can be seen in the figure, mean dip estimates for 10 of the 13 models do not differ from 90°. Figure 11b shows the frequency distribution of the dip estimates across all models; 62 % of the responses fell within 10° of 90°. This suggests that while the variability is greater with the models than with the pictures, observers still exhibit a prior to report regions as extending straight back.

Are the visual inferences of dip angles based on memory for the object? To answer this, we examined the percentage of trials in which participants reported perceiving the layer as they had originally. Participants reported perceiving their original perception in 62 % of the trials and reported a new orientation in 38 % of the trials.^4^ This proportion is quite similar to the proportion of trials in which subjects reported forms going straight into the photographs in Experiment 2. A binomial test revealed that the proportion of participants who reported their original answer was greater than expected by chance [*p* < .001 (2-sided)].^5^

A strict perception view would expect participants to revert to their original perception in 100 % of the trials, whereas a strict memory or knowledge hypothesis would expect participants to always switch their report based on new knowledge. Neither of these is supported by our data. A one-sample *t* test comparing the mean proportion of trials in which participants reported their original perception is different both from 100 % staying with the original perception [*t*(39) = −10.1, *p <* .001] and from 0 % staying with their original perception [*t*(39) = 17.4, *p <* .001].

**Fig. 12:**
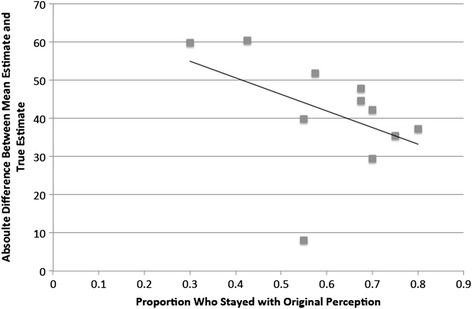
A scatterplot of the relationship between the proportion of trials in which participants stayed with their original response and the absolute difference in degrees between the mean estimate and the true estimate for each model

Which factors lead to a switch in participants’ reports? In a follow-up analysis, we found that switching was more likely when there was a large difference between the actual dip angle (i.e., the correct answer) and observers’ initial estimate. Figure 12 shows a scatterplot of the relationship between the proportion of trials in which participants held their original perception and the absolute difference between the correct answer and the mean dip estimate. The two models where the correct answer is straight down are removed from this analysis. As can be seen, there is a negative relationship; observers were less likely to stick with their original estimate when there was a larger difference between the correct answer and their initial report. This is perhaps the result of some demand characteristics and conditions that lead to switching requiring further examination.

In sum, the prior to report regions as extending straight back often occurs despite knowledge that it does not. This suggests that this prior is similar to other examples of amodal perception, such as the Michotte Triangle.

## Experiment 4

It is possible that this effect hinges on demand characteristics; when observers are asked about the inside of an object that they have no information about, they have a response bias to say the region extends straight back. To address this, we asked if participants would be willing to make a commitment about the location of an object without any visual support.

Observers viewed pictures of the outsides of objects that were likely to contain different objects inside (e.g., a toy box, suitcase, grocery store) but where no visual information about the “inside” object was provided. For each picture, observers were asked if a specific object was inside (Are Legos inside this toy box? Is a toothbrush inside this suitcase? Is a cashier inside this store?). They were then told the specific object was inside and then asked to indicate where it was. If participants are biased to report that any structure is straight back from a surface, then we would expect the distribution of responses to clustered around 90°. If there is no bias, then we would expect their estimates to be widely distributed.

### Methods

#### Participants

Participants were 20 Temple University undergraduates (12 females) fulfilling a requirement for an introductory psychology course.

#### Stimuli

**Fig. 13:**
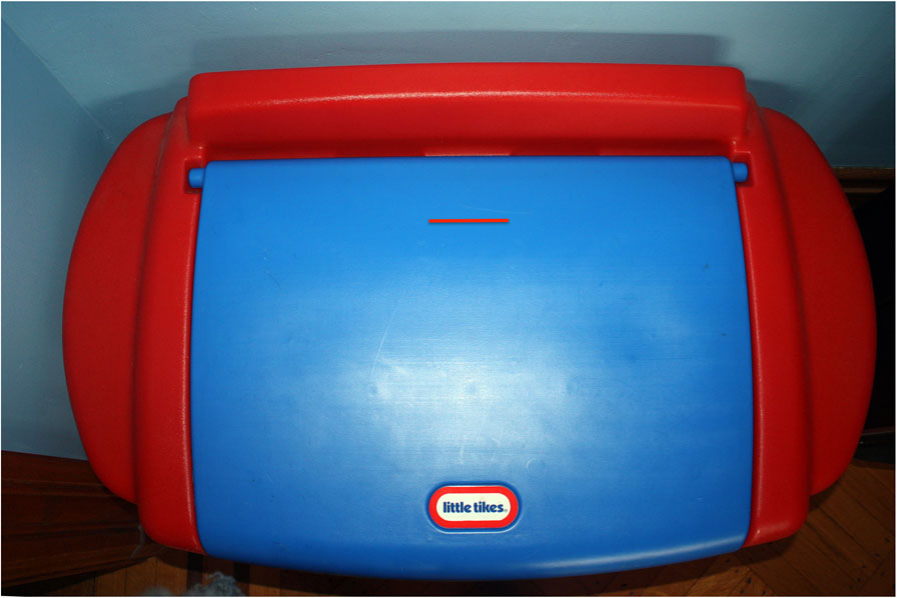
The toy box stimulus used in Experiment 4. Participants were asked about the presence of Legos inside the toy box and were told that the Legos were indeed inside and asked to place a bar on the line and indicate where they guessed the Legos would be

The stimuli consisted of 16 color photographs of common objects or settings. These included 1 practice picture and 15 experimental pictures. Four were from the set used in Experiments 1 and 2, and 11 were new. The new stimuli consisted of pictures of the outsides of objects where another object could be inside: toy box, suitcase, grocery store, oven, glue stick tube, board game, house, refrigerator, wall, piggy bank, and pregnant woman’s torso. For each picture, we asked whether a specific object was inside. The stimuli-object parings fell into two classes: parings for which (a) the object was likely inside but its location was unknown (i.e., a cashier inside a store) and (b) it was impossible to tell whether the object was inside or not (i.e., Legos inside a toy box). As shown in Fig. 13, each image contained a red line that served as a starting point for making the location estimate. The images were approximately 24 × 36 cm in size.

#### Apparatus

Stimuli were presented on a 21-inch Mac desktop computer (Apple, Cupertino, CA, USA). Participants were seated approximately 45 cm away from the screen.

#### Procedure

The procedure was similar to that in Experiment 2, with the following exceptions. Observers were shown 16 pictures. For each, they were asked whether a specific object was inside and selected one of the following answer choices and then indicated their confidence: (1) yes, the object is inside and I can use the bar to show you where it is; (2) yes, the object is inside but from the picture I cannot tell where it is; (3) from the picture I cannot tell whether the object is inside; or (4) no, the object is not inside. If Choice 1 was selected, they used the bar with inclinometer to indicate the location of the object. After they had made their selections for all pictures, they were told that the object was inside and asked to guess about the location by orienting the bar.

### Results and Discussion

Participants were fairly confident in their responses; mean confidence was 4.0 (SD 1.0). In 84 % of the trials, participants indicated that they could not tell the location of the object from the picture (either by selecting cannot know the location or cannot know at all whether the object is there). Estimates of location were given in 11 % of the trials, and observers reported that the object was not inside in 5 % of the trials. Clearly, participants are not willing to make an estimate without perceptual support.

**Fig. 14:**
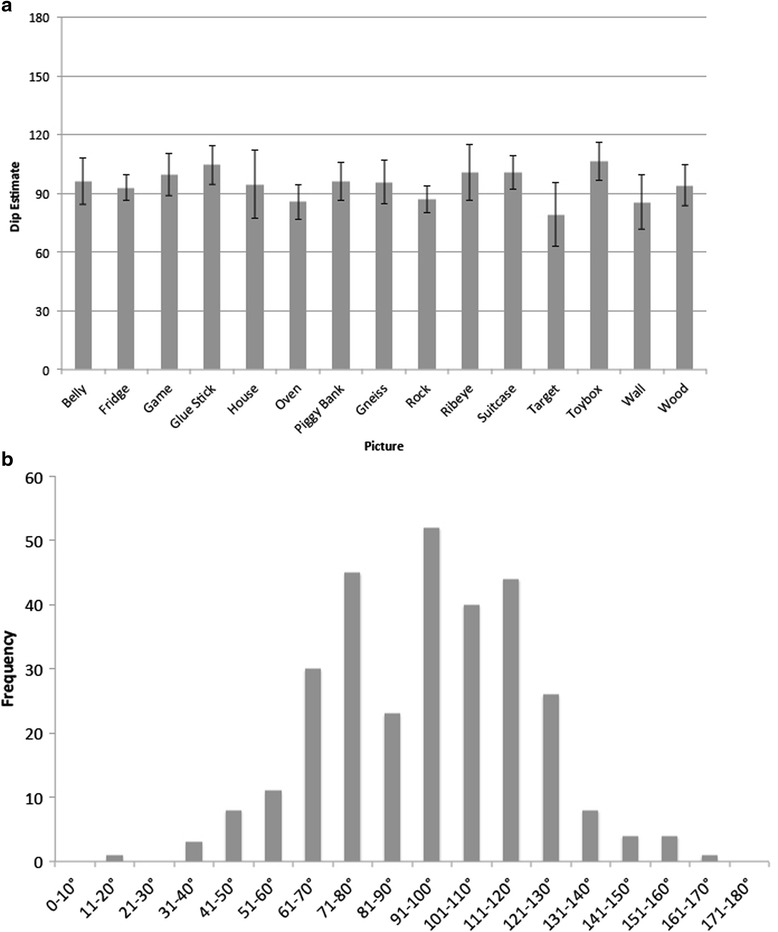
**a** The mean dip estimates in Experiment 4. **b** Frequency distribution of responses that fell within angle bins in Experiment 4

Mean dip estimates for each picture are shown in Fig. 14a, and the distribution of responses is shown in Fig. 14b (collapsing across trials in which participants were required to make an estimate and the trials in which they chose to make one). As can be seen from the figure, the average of the estimates is close to 90°. However, the distribution of responses does not cluster within 10° of 90°. Indeed, only 25 % of the responses fell within 10° of 90°; observers’ estimates ranged from 16° to 160°.

These findings suggest that the prior observed in the previous experiments cannot be explained by participants’ response biases. When there is no obvious perceptual information related to an object at the surface, observers do not make commitments about its interior location.

## General Discussion

How do observers make inferences about the interior 3D structure of objects from information visible on the surface? We found that observers appear (a) unaware that the 3D structure cannot be inferred from a cross-section and (b) to have a *prior* to infer that a layer on a cross-section at the surface extends straight back into the object. This was true for both photographs of single surfaces (Experiments 1 and 2) and for 3D objects (Experiment 3). Experiment 2 showed that, when provided with two sides and thus enough evidence to infer how the region extends into the object, observers can solve the task. However, in the absence of sufficient evidence (i.e., only a single cross-sectional view), perception appears to be governed by this prior. Experiment 3 suggests that this prior is not easily influenced by knowledge of the object, and thus appears to be perceptual in nature, similar to amodal completion. Experiment 4 showed that participants’ reports are unlikely to be the result of a response strategy to say “straight in” for anything that is inside an object, as this prior was not evident in the absence of visual information about the interior structure. Finally, while all observers showed a similar prior, observers who scored high on a separate measure of inferring 3D structure from multiple sides were more likely to recognize that inferring the 3D structure was not possible from a single cross-section alone.

Humans can show significant errors in reasoning about physically transforming objects (McCloskey, 1983; Pani, 1997). We argue that the prior observed here is *perceptual* in nature rather than a function of the observer’s knowledge or general reasoning based on findings across the experiments. In Experiment 3, even when participants were shown how a region extended into the object (i.e., the correct answer), they tended to return to the original report when that information (i.e., the correct answer) was no longer visible. This observation suggests a clear similarity to amodal perception (Kanizsa, 1979; Michotte et al., 1964), where completion occurs in spite of participants’ knowledge of the object. These reports cannot be accounted for by demand characteristics or general beliefs about where things are inside objects; in Experiment 4, participants did not make commitments about the orientation of the interior region in the absence of visual information at the surface. Finally, in Experiments 1 and 2, we found no evidence that skill in reasoning about spatial problems was related to the prior; individuals’ ability to mentally simulate complex events (e.g., object rotation or view from a new perspective) did not appear to be related to their reporting structures projecting straight into a volume. Indicating that the prior is not the product of using spatial reasoning to infer the spatial relationships between the cross-sectional plane and the intersecting interior plane.

### Is This a Form of Amodal Completion?

The observation that the prior is not easily influenced by knowledge of the object’s form shows a similarity to other examples of amodal completion, such as the Michotte triangle (see Fig. 9). This suggests a prior that supports a visual inference to uniquely extend volumetric representations into regions where the form is not visible. We propose this as an example of *amodal extension in depth*. A number of studies have documented amodal completion in depth (Gerbino & Zabai, 2003; Palmer, Kellman, & Shipley, 2006; Tse, 1999; Van Lier and Wagemans, 1999). However, these are cases of amodal *interpolation*, where contour or surface completion links two visually specified regions. The extension reported here represents *extrapolation*. Extrapolation can occur in both amodal (Kanizsa, 1979) and modal displays (Shipley & Kellman, 2003).

Modal extrapolation is limited in extent (Kanizsa, 1979). So, too, the phenomenon reported here may show similar limits. Our evidence shows that the extension of a region into three dimensions governed by a prior continues from the immediate surface back into the object. We do not assess the shape of the extension or its presence deep inside the volume. We note anecdotally that the raisins in Fig. 2 do not appear to be raisin tubes. Here it is possible that the visual system continues a curved surface to close the raisin volume. How deeply a single surface would be extrapolated is an open question, and these limits may be examined in further research.

Importantly, the extrapolation we report here does not begin from a visually specified edge and continue from that edge; here extrapolation begins at an edge on a surface and proceeds perpendicularly to that surface into regions that are not visible. This is reminiscent of illusory contours that appear perpendicular to line ends (Kennedy, 1978). Perhaps there is a surface analogue to end-stopped cells (Heitger, Rosenthaler, Von Der Heydt, Peterhans, & Kübler, 1992) that play a role in binding faceted surfaces into single objects. When a surface ends, our data suggest that, rather than representing this region as dimensionless, the visual system has a prior to represent, perhaps automatically, an orthogonal surface at the boundary. Although these suggestions are highly speculative, the critical finding invites a new question: *How does* the visual system construct a representation of 3D form that extends into a volume? Our aim is not to offer a new theory, but instead to suggest that the types of visual inferences reported here need to be explained by any theory of visual completion. We document the systematic nature of these types of inferences with the hope of bringing this research to the attention of the community.

### How Does the Visual System Represent 3D Interior Structures?

The present work does offer a few hints that may help develop a full model of this process. As has been found in research on other shape-from-X cues, the visual system appears to follow some fundamental constraints that allow a unique solution when there is the potential for many-to-one mappings. For example, a rigidity constraint allows the visual system to recover shape from motion patterns (Wallach & O’Connell, 1953; see also Sperling, Landy, Dosher & Perkins, 1989; Mather, 1989; Richards et al., 1987), and closure and smoothness constraints allow inferences of 3D shape from 2D silhouettes (Richards et al., 1987). Here the finding that, for single surfaces, observers tend to report the interior forms as extending straight back into the object suggests a visual constraint that may help solve the inference problem for multiple potential surfaces. The present research suggests a *default constraint*, or *prior*, where interior structures extend orthogonally to exterior surfaces (unless there is contradictory evidence). Thus, in the cases in Experiments 2 and 3, where two cross-sections were visible, the interior structures may result from simultaneous extrapolations from two cross-sectional surfaces.

It is perhaps worth emphasizing that the observed prior, to infer regions as extending straight back, is not trivial. The system could have no prior, with wide-ranging estimates of interior structures across or within individuals. Alternatively, no structure could be seen when there is insufficient evidence to determine the 3D form. If indeed the phenomenon is perceptual in nature, *seeing* a structure as extending straight down may be analogous to seeing the shortest path in apparent motion; it may be “sensible” and minimize error between guess and reality, but nonetheless it requires a theoretical explanation.

### Relationship to Science, Technology, Engineering, and Math (STEM) Education

This research project arose from a collaboration that encompassed cognitive science and the geosciences (Shipley, Tikoff, Ormand, & Manduca, 2013). The illusion that geological features extend straight back into an outcrop is exciting because it may be revealing about previously unrecognized underlying visual processes; however, such illusions are a practical challenge for STEM education. The need to think about the interior 3D structure from surface views is common in medicine (e.g., ultrasounds, CT and MRI scans; Chariker, Naaz, & Pani, 2011; Hegarty, Keehner, Cohen, Montello, & Lippa, 2007; Wu, Klatzky, & Stetten, 2010, 2012), mathematics (e.g., conical sections), crystallography (e.g., a sectional view of atoms in a solid), biology (thin sections; LeClair, 2003), dentistry (Hegarty, Keehner, Khooshabeh, & Montello, 2009), and the geosciences (Kali & Orion, 1996).

It is important that teachers recognize that students have a tendency to infer that regions in a cross-section extend straight back into objects. Experts likely avoid simple versions of this error, but see Shipley and Tikoff (2016) for an example that we interpret as a case where experts may be making this error. Perhaps students who are aware of the potential to make this error will be more likely to succeed and continue in the discipline; the relationship between understanding 3D relationships and recognizing the ambiguity we found in Experiment 2 is consistent with such a hypothesis. If so, developing interventions that help students overcome this error may contribute to expanding STEM education. One potential intervention may be training an understanding of the relationship between cross-sections and 3D structures. Naaz, Chariker, and Pani (2014) found that students given sufficient training could accurately go back and forth between 3D whole-brain representations and 2D neuroanatomical cross-sections. The role of learning about 3D structures and their cross-sections is an important direction for future research in this area.

## Conclusions

Participants tend to perceive regions visible in a cross-section as being 3D and extending back into the object at 90°. We suggest that this inference represents a form of amodal completion, *amodal extrapolation*, which has previously been unrecognized. We argue that this finding reveals constraints within the visual processes that serve perception of objects. The aim of this paper is to bring this phenomenon to the attention of the perception community and to illustrate the important connection between basic psychological research and STEM practice and education. By working in an interdisciplinary group on students’ struggles to comprehend complex spatial information, one reveals aspects of the human mind that cognitive science has yet to recognize.

## Footnotes

1 Here we borrow a term from the earth sciences: technically *dip*, or *dip angle*, is the steepest angle of descent of a feature relative to a horizontal plane. Here we use the term to refer to the angle of descent into an object relative to the surface plane of that object.

2 This was true when the analysis included all dip estimates, and when the analysis was restricted to only the dip estimates reported when the picture was accurately identified.

3 We note that this error is higher than the angular error reported by Kozhevnikov and Hegarty (2001) (33.2, SD 23.3), but we have no obvious explanation for the elevated error.

4 There were two models for which the layer extended straight down. When those models were removed from this analysis, participants still reported the orientation of the layer as having the one they had originally reported in 61 % of the trials.

5 This was the case both when all models were entered into the analysis and when the two models where the layer extended straight down were excluded.
